# AAV-mediated BDNF and GAS6 muscle delivery delays disease onset in SOD1^G93A^ ALS mice

**DOI:** 10.1038/s41434-025-00577-y

**Published:** 2025-11-13

**Authors:** Yicong Le, Gongjie Liu, Shenzhe Wu, Marialaina Nissenbaum, Alexander W. Kusnecov, Philip Furmanski, Raymond B. Birge, Renping Zhou

**Affiliations:** 1 Department of Chemical Biology, Ernest Mario School of Pharmacy, Rutgers, The State University of New Jersey, Piscataway, NJ USA; 2 Panathera Inc., Piscataway, NJ USA; 3 Department of Psychology, School of Arts and Science, Rutgers, The State University of New Jersey, Piscataway, NJ USA; 4 Rutgers Cancer Institute of New Jersey, New Brunswick, NJ USA; 5 Environmental and Occupational Health Sciences Institute, Piscataway, NJ USA; 6 Department of Microbiology, Biochemistry, and Molecular Genetics, Rutgers School of Biomedical and Health Sciences, Newark, NJ USA

**Keywords:** Neurological disorders, Gene expression

## Abstract

Amyotrophic Lateral Sclerosis (ALS) is a fatal neurodegenerative disease, with limited treatments. Gene therapy offers an alternative strategy for treating a large portion of ALS patients, however, the disparate genetic alterations in ALS complicate the development of gene therapies. Tyrosine receptor kinase B (TRKB) and Tyro3 receptors are highly expressed in mouse spinal cord motor neurons, suggesting that their ligands, brain-derived neurotrophic factor (BDNF) and growth arrest-specific 6 (GAS6), respectively, are crucial for neuronal survival. In this study, we tested whether genetically induced and muscle tissue-specific expression of such survival-enhancing ligands would ameliorate symptom development in the SOD1^G93A^ ALS mouse model. The therapeutic vectors (AAV-P_*mus*_7-HuBDNF-teLuc or AAV-P_*mus*_7-HuGAS6), or a control vector (AAV-P_*mus*_7-teLuc) were injected intravenously via the retro-orbital route and intramuscularly into the hindlimb skeletal muscle of six-week-old mice. Treatment with the therapeutic vectors delayed disease onset and slowed progression in both male and female mice. Interestingly, a sex-specific response was observed, with female mice benefiting more from the treatments than males. Lumbar motor neuron survival was more sustained in the therapeutic vector-treated group compared to control vector group. No statistically significant extension of lifespan was observed in the treated groups.

## Introduction

Amyotrophic lateral sclerosis (ALS) is a severe neurodegenerative condition characterized by the progressive degeneration of upper motor neurons in the motor cortex and lower motor neurons in the brainstem and spinal cord. ALS has an incidence rate of about 2 per 100,000 people each year and a prevalence of 6-9 per 100,000 individuals worldwide [1, 2], leading to a lifetime risk estimated at approximately 1 in 350 [3]. Evidence suggests that the incidence of ALS is rising [1], possibly due to shifting population demographics and advancements in clinical services that enhance diagnostic accuracy. A family history of ALS (fALS) is found in 5-10% of those affected, many mutations have been identified that have been related to ALS; editing those genes is one potential strategy for gene therapy of fALS, but the large number of genes involved would greatly complicate the treatments [4–8]. In 90-95% of ALS patients, however, the disease is considered sporadic (sALS), most of which do not have known causes [5]. An alternative is to attempt to protect affected neuronal tissue by increasing the local bioavailability of trophic factors [9]. Based on our analysis of RNA seq data from ALS-affected motor neurons [10], we identified several receptors for neurotrophic factors highly expressed in these cells which could be targets for such a therapeutic intervention.

Brain-derived neurotrophic factor (BDNF) belongs to the neurotrophin family; it was found to support the survival and growth of cultured spinal motor neurons isolated from rodents via activation of tyrosine receptor kinase B (TRKB) [11, 12]. Early clinical trials tested BDNF administered subcutaneously and intrathecally, phase I/II trials indicated that both delivery methods were well-tolerated, with intrathecal delivery showing fewer adverse effects [13, 14]. But the phase III trial for systemic BDNF administration did not yield clinically significant benefit [15]. Similarly, other phase III trials for intrathecal BDNF delivery also failed to demonstrate beneficial effects [14, 16]. One limitation in these trials is BDNF’s poor tissue permeability, potentially reducing the effective dose reaching target neurons. To overcome this, alternative approaches have been explored, such as intrathecal injection of cells that secrete BDNF. In SOD1^G93A^ mice, human umbilical cord mesenchymal stem cells differentiated into motor neurons and overexpressing BDNF, improved motor control and extended lifespan, compared with mice receiving cells not expressing BDNF [17]. However, not all cell-based approaches have been successful; for instance, neural progenitor cells overexpressing BDNF did not enhance motor abilities or survival when implanted into the spinal cords of SOD1^G93A^ mice [18], highlighting the importance of cell type and transplantation method in determining the success of such therapies.

Growth arrest-specific 6 (GAS6) is a secreted protein that interacts with Tyro-3, which belongs to the TAM receptors family (Tyro-3, Axl, and Mer) [19]. This GAS6/TAM signaling pathway is crucial for several physiological functions, including cell migration, adhesion, growth, and survival [20]. Successive studies have revealed roles for GAS6/TAM signaling in promoting hemostasis [21], inhibiting inflammation [22], and slowing tumor progression [23]. There is also evidence linking GAS6 to autoimmune processes within the central nervous system (CNS) [24, 25]. GAS6 and its TAM receptors are expressed in the brains of both embryonic and adult rats, with their expression levels correlating with synaptogenesis in cerebral tissues; GAS6 exerts proliferative and antiapoptotic effects on various CNS cell types, including hippocampal and cortical neurons, Schwann cells, and oligodendrocytes [25–28]. Research by Binder and colleagues demonstrated that the absence of GAS6 impairs remyelination efficiency following a demyelinating injury, as seen in a cuprizone-induced model [29]. Recent studies have shown that GAS6 promotes microglial entry, reduces neuroinflammation in hemorrhagic mice, and alleviates white matter injury after ischemic stroke [30, 31]. Furthermore, administering recombinant human GAS6 to the corpus callosum in this model enhanced myelin repair [32]. These findings suggest that the GAS6/TAM system plays a critical role in regulating the survival of neuronal and glial cells, particularly those involved in myelination, and in controlling the innate immune response.

In this study, we delivered BDNF and GAS6 into muscle cells of ALS model mice using AAV vectors with a strong muscle-specific promoter. Secreted BDNF and GAS6 from muscle cells were able to support interacting motor neuron survival, postpone disease onset and alleviate the disease symptoms.

## Materials and methods

### Vector plasmid construction

pAAV plasmids were generated using the pAAV-CMV-teLuc backbone which was constructed by substituting the eGFP gene from the pAAV-CMV-eGFP (Addgene Cat. 67634) with a teLuc gene PCR fragment, utilizing pcDNA3-teLuc c-myc (Addgene Cat. 100026) as the template. The pAAV-P_*mus*_3-teLuc construct contains a sequence -744 to -958 relative to the first exon of human *DES* gene (hu DES enhancer) and combined with one 2R5S (murine Mck) enhancer [33] and the Mck core promoter [34]. Mature HuBDNF sequence [35],tagged with the Hemagglutinin (HA) sequence, was ligated downstream of the promoter, followed by a wildtype IRES sequence and reporter gene teLuc [36] to make pAAV-P_*mus*_3-HuBDNF-teLuc. pAAV-P_*mus*_7-HuBDNF-teLuc was constructed by replacing the promoter region of pAAV-P_*mus*_3-HuBDNF-teLuc construct with a *sk-CRM*4 enhancer element and a murine *Des* promoter [37]. In pAAV-P_*mus*_7-GAS6, human GAS6 cDNA (MN_000820.3) tagged with the Flag tag was ligated in place of the BDNF sequence.

### Cell culture and Western blot

HEK293T cells (Takara Bio, 632180) were cultured in growth medium composed of DMEM (4500 mg/L Glucose, Gibco) supplemented with 10% FBS (Gibco), 1x MEM non-essential amino acid (MEM NEAA, Gibco), 1 mM Sodium Pyruvate, 100 U/mL penicillin-streptomycin (Gibco), and 1.5 g/L Sodium Bicarbonate. C2C12 muscle cells (ATCC, CRL-1772) were maintained in DMEM (4500 mg/L Glucose, Gibco) supplemented with 10% FBS, and differentiated in DMEM (4500 mg/L Glucose, Gibco) supplemented with 2% horse serum. For western blot, C2C12 cells were infected with AAV vectors at MOI 10,000 on differentiation day 4 in a 100 mm culture dish. 4 days after infection, cell pellet and culture medium were collected and tested for expressed and secreted recombinant protein. Cell pellets were lysed in NP-40 (50 mM Tris-HCl, 150 mM NaCl, and 1% NP-40) with 1x proteinase inhibitor (Sigma P8340). Cell lysates were then centrifuged at 18,000 × *g* for 20 min and supernatants were collected and boiled with 5% of β-mecaptoethanol and 4x Laemmli buffer. Protein concentrations were determined with Pierce BCA Protein Assay Kit (Thermo Scientific 23225). Secreted recombinant proteins were concentrated by immunoprecipitation following the manufactures’ instruction (Anti-DYKDDDDK G1 Affinity Resin, GenScript L00432; Pierce Anti-HA Magnetic Beads, Thermo Scientific 88836). Recombinant huBDNF was revealed with anti HA-Tag (C29F4) Rabbit mAb (Cell Signaling 3724) and Goat-anti-rabbit secondary (Thermo Scientific #32460). Recombinant huGAS6 was revealed with anti-Flag Mouse mAb (Sigma F1804) and Goat-anti-mouse secondary antibody, HRP (Thermo Fisher Scientific #62-6520).

### Virus production and purification

AAV.PHP.eB was produced using Gibco AAV-Max Helper-Free AAV production System (A51217). In brief, Viral Production Cells (VPC) were cultured in the Viral Production medium. When reaching 3 million/mL concentration, VPCs were transfected with recombinant pAAV, pHelper (Takara Bio #6234), and pUCmini-iCap-PHP.eB [38] using the AAV-Max transfection kit. 70–72 h post transfection, cells were lysed by adding AAV-Max Lysis buffer, and cell debris were removed by centrifuging at 3,500 × *g* for 30 min at 4 °C. Virus particles in the supernatants were precipitated with polyethylene glycol (PEG) 8000 solution (8% PEG and 0.5 M NaCl final concentration) and centrifuged at 5000 × *g* for 1 h at 4 °C. Concentrated AAVs were purified by two rounds of CsCl gradient ultracentrifugation (38,000 × *g*, 18 h, 20 °C). Virus titers were determined by qPCR with the following primers:

CRM4+: 5’-AGCCGTCTCCCTAGCAGCCA-3’.

DES-: 5’-TCCTCAGGACATCGGGTCCTAGA-3’.

teLuc+: 5’-CCGGCTACAACTTGAGTCAAGTCC-3’.

teLuc-: 5’-CTTCATACGGGATGATGACATGGATGTC-3’.

### Immunostaining

Mouse spinal cords were obtained by the hydraulic extrusion protocol [39]. In brief, segment L1-L6 lumbar spinal cord was dissected; Isolated lumbar spinal cord tissues were soaked in 4% paraformaldehyde (PFA) (Thermo Fisher AAJ19943K2) for 24 h followed by immersion in 15% and 30% sucrose solution until they settle down to the bottom of the tube. A total of 100 sections were collected per animal across 10 slides. Slides containing comparable anatomical regions were selected for staining, and motor neurons were quantified. The average motor neuron count per ventral horn was calculated and presented. Sections were treated with prewarmed (60 °C) antigen retrieval buffer (10 mM Sodium Citrate, pH 6.0) for 30 min before primary goat ChAT antibody (Sigma Ab144p, 1:100) incubation for 18 h followed by peroxidase conjugated secondary antibody (Abcam AB214881, 1:1000). DAB (Abcam ab64238) was used as the chromogenic reagent. ChAT positive cells in the ventral horn were counted.

### ELISA

Heart, triceps branchii (TB), quadriceps (Quad) muscles, and brain tissues were dissected from both male and female animals and lysed in NP-40 buffer (1% NP-40, 150 mM NaCl, and 50 mM Tris-Cl, pH8) supplemented with 1% (v/v) Proteinase Inhibitor Cocktail (Sigma P8340) for 30 min on ice, supernatants were collected after centrifuge at 1800 g for 20 min. The supernatants were used for recombinant protein detection by the Human BDNF Elisa Kit (Sigma RAB0026) and DYKDDDDDK-tag Detection Elisa kit (Cayman No. 501560).

### Transgenic mice

Transgenic mice with the G93A human SOD1 mutation (B6SJL.SOD1-G93A) were originally obtained from the Jackson Laboratory (Bar Harbor, ME, USA). Colonies were expanded by crossing with C57BL/6 J females. The offspring were genotyped by PCR of DNA extracted from tail tissue. Animal numbers of each experimental group were determined by resource equation approach (group comparison, one-way ANOVA). The following experimental groups of mice were used: male SOD1^*G93A*^ mice injected with AAV-P_*mus*_7-teLuc (male control, *n* = 11), AAV-P_*mus*_7-BDNF-teLuc (male BDNF, *n* = 11) or AAV-P_*mus*_7-GAS6-teLuc (male GAS6, *n* = 11); female SOD1^*G93A*^ mice injected with AAV-P_*mus*_7-teLuc (female control, *n* = 13), AAV-P_*mus*_7-BDNF-teLuc (female BDNF, *n* = 10) or AAV-P_*mus*_7-GAS6-teLuc (female GAS6, *n* = 11), each group of animals were allocated based on weight and litter to ensure similar group composition. Transgenic mice were injected at 6 weeks of age with a total of 2 × 10^12^ virus genomes (vg) both intravenously (1 × 10^12^ vg, retro-orbital) and intramuscularly (5 × 10^11^ vg) into the quadriceps of each hindlimb. Investigators were not blinded to the group allocation and when assessing the outcomes.

### Behavioral studies

#### Disease onset

The onset of disease of the transgenic mice was determined by bodyweight (BW) and neurological score (NS) change. Animals were first examined weekly after virus injection, and then daily after animals reached NS 2. NS was determined as described [40]. The NS onset was determined as the age at which the first signs of motor impairment were manifest: the inability of the mouse to stand upright and completely extend its hindlimbs, and also the appearance of a slight tremor in one or both hind limbs while the mouse was lifted by its tail. The BW onset was determined as the age when body weight starts to decrease steadily.

#### Lifespan

Mice of all experimental groups were maintained in the colony until they reached the experimental endpoint. The age of death was defined as an animal’s inability to right itself within 15 s after being placed on its side. The date the animal became moribund represented the experimental endpoint and the date of death.

#### Locomotor performance by Rotarod

Mice were tested for their ability to maintain balance on a rotating rod (7 cm diameter) at 18 rpm by using a Rotarod apparatus (Columbus Instruments, Ohio), starting at 64 days of age. Each test session consisted of 3 trials (arbitrarily 3 min/trial), and each trial was separated by a 5-min rest period. Performance time for each trial was recorded as the longest time the mouse could remain on the rod without falling. Measurements were performed each week until the animals fall off the rotarod for less than 5 s.

## Results

### Recombinant AAV vector construction and neurotrophic factor expression

To identify which neurotrophic factors might promote survival of the spinal motors neurons, we analyzed receptor tyrosine kinase (RTK) expression in adult mouse spinal cord using RNA-seq profiling data of spinal motor neuronss in ALS mice [10]. Our results showed that Ret, Ntrk2, and Tyro 3 are among the top RTKs which have higher expression levels in these cells (Table 1). The Ret receptor ligand glial-derived neurotrophic factor (GDNF) has been shown to have neuroprotection and extend life span in mouse ALS models; however, there is no published study on AAV delivery of Ntrk2 receptor ligand BDNF in ALS [41]. The role in ALS of GAS6 which is the Tyro3 receptor ligand has not previously been reported in ALS, either. Although their RPKM values did not significantly differ between groups, we highlighted these genes due to their established neuroprotective roles and their potential to counteract ALS-related neurodegeneration.

**Table 1:** Top potential NTR receptors expressed in adult mouse motor neurons.

Gene Symbol	Name	W1-RPKM	W2-RPKM	G85R1-RPKM	G85R2-RPKM	Ligand
*Receptor Tyrosine Kinase*						
**Fgfr1**	fibroblast growth factor receptor 1	661.47	739.12	489.63	502.95	**FGF1**
**Ret**	ret proto-oncogene	144.03	164.85	109.99	118.13	**GDNF**
**Ntrk2**	Neurotrophic tyrosine kinase, receptor, type 2	31.83	34.58	31.95	34.19	**BDNF**
**Flt3**	FMS – like tyrosine kinase 3	24.85	25.07	25.39	27.13	**FLT3L**
**Epha10**	Eph receptor A10	22.76	24.32	15.29	16.62	**EFNA**
**Ephb6**	Eph receptor B6	19.51	23.58	19.12	22.24	**EFNB**
**Tyro3**	TYRO3 protein tyrosine kinase 3	16.16	17.47	16.36	16.75	**Gas6/PROS1**
**Ntrk3**	Neurotrophic tyrosine kinase, receptor, type 3	14.68	16.36	13.56	12.76	**NT3**
**Ephb1**	Eph receptor B1	12.35	13.65	9.31	9.44	**EFNB**
**Fgfr2**	fibroblast growth factor receptor 4	9.37	8.29	7.19	5.88	**FGF1**

Analysis of receptor tyrosine kinase of neurotrophic factors (NTF) expression based on deposited RNA-seq data [5] in adult mouse motor neuron.

*W* wild type, *G85R* a *SOD1* mutant, *RKPM* reads per kilobases per million reads to normalize gene size and sequencing depth.

In this study, we aimed to express trophic factors BDNF and GAS6 in muscle cells to support innervated motor neuron survival in ALS model mice. Our earlier study showed that the AAV variant PHP.eB had higher expression level in muscle cells and tissues than AAV2-MTP and AAV6 [42], so this AAV capsid was used in this study.

**Fig. 1:**
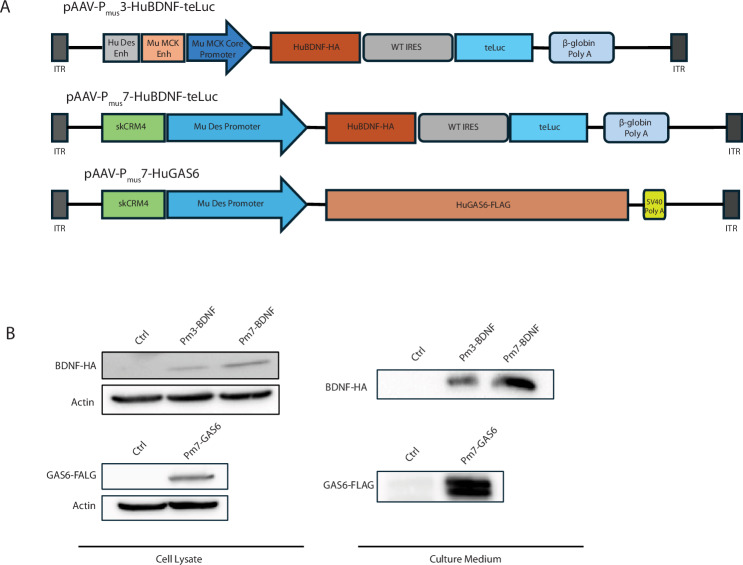
Virus construct structure and in vitro trophic factor expression. AAV vectors were designed and constructed based on previously published muscle specific promoters and enhancers, as well as using regulatory elements from genes which are highly expressed in muscles. Trophic factors BDNF and GAS6 were then cloned into the selected vectors. Virus constructs were tested in vitro for trophic factor expression before delivery into animals. **A** AAV construct diagram of AAV-P_*mus*_3-huBDNF-teLuc, AAV-P_*mus*_7-huBDNF-teLuc, and AAV-P_*mus*_7-huGAS6. P_*mus*_: muscle-specific promoters; Mu: murine; Hu: human; Mck: muscle creatine kinase gene; Des: desmine gene; CRM: cis-regulatory modules; IRES: internal ribosome entry site; teLuc: a modified luciferase; Poly A: poly-adenylation signal. **B** Western Blot of control virus AAV-P_*mus*_7-teLuc (Ctrl), AAV-P_*mus*_3-huBDNF-teLuc (Pm3-BDNF), AAV-P_*mus*_7-huBDNF-teLuc (Pm7-BDNF) and AAV-P_*mus*_7-HuGAS6 (Pm7-GAS6) expression in differentiated C2C12 cells. Viruses were infected on differentiation day 4 and cell lysate and culture medium were collected 4 days after infection.

We designed two constructs based on previously published muscle specific promoters and enhancers, as well as using regulatory elements from genes that are highly expressed in the muscle tissue. The P_*mus*_3 promoter was a combination of a human DES enhancer, a 2R5S enhancer [33] and a murine Mck promoter (Fig. 1A). The Pmus7 promoter was adopted from Sarcar et.al. which contains a skCRM4 element and a murine Desmin promoter (Fig. 1A). BDNF-HA were cloned into the AAV-P_*mus*_3 and AAV-P_*mus*_7 vectors together with te-luciferase (teLuc) as a reporter gene [36] to test protein expression in vitro.

Differentiated C2C12 myotubes were infected with AAV-P_*mus*_7-teLuc (control virus), AAV-P_*mus*_3-BDNF-teLuc, and AAV-P_*mus*_7-BDNF-teLuc (MOI 10,000). At 4 days post infection, cell lysates and culture medium were collected for the detection of BDNF expression. BDNF proteins in the medium were first concentrated using anti- HA magnetic beads. Western blots showed that both AAV-P_*mus*_3-HuBDNF-teLuc and AAV-P_*mus*_7-HuBDNF-teLuc vector can express the recombinant protein in C2C12 myotubes, however, AAV-P_*mus*_7-HuBDNF-teLuc vector had higher expression levels of BDNF, which is consistent with the reporter gene assay (Fig. 1B). Thus, we chose to use the AAV-P_*mus*_7-HuBDNF-teLuc as the potential therapeutic vector.

To further elucidate the muscle tissue specificity of these vectors, recombinant AAV viruses with the reporter gene te-Luciferase under the transcriptional control of the pan-cell type promoter CMV or muscle- specific promoter P_*mus*_7 were packaged with AAV2 which infects HEK 293 T cells well or PHP.eB, respectively. After transduction of HEK 293 T cells, robust luciferase activity from the AAV2-CMV-teLuc infected cells were observed, whereas the cells infected with AAV2-P_*mus*_7-teLuc showed limited expression compared to the CMV group (Fig. S1A). In contrast, with PHP.eB capsid, P_*mus*_7 directed higher te-Luciferase expression than CMV in C2C12 muscle cells (Fig. S1B), which indicated the promoter specificity of this vector.

GAS6-FLAG was then cloned into the pAAV-P_*mus*_7 vector. The protein expression and secretion were also validated by transducing the differentiated C2C12 myotubes followed by Western Blot. The secreted GAS6 in the culture medium was first concentrated by anti-FLAG affinity resin. AAV-P_*mus*_7-HuGAS6 also showed protein expression both in the cell lysates and the culture medium (Fig. 1B).

### The effects of BDNF and GAS6 expression in skeletal muscle on disease onset

After we confirmed that our AAV vectors were able to successfully express the trophic factors in the muscle cells in vitro, we tested their efficacy in vivo in the ALS mouse model. Directly injecting the virus into skeletal muscles was considered as potentially more efficient for muscle specific expression. In our earlier studies, a re-inducible muscle-specific expression vector, packaged in the same capsid, was administered intravenously to mice, and the expression level of the reporter gene (te-luc) was tracked following repeated inductions. After 18 weeks, reporter gene expression remained sustained following the third induction, indicating long-term expression after systemic injection [42]. In contrast, the same vector was administered intramuscularly to a separate group of mice. After the second round of induction, expression levels decreased by 42%, and by 17% after the third induction, compared to the initial induction (data not shown). To achieve the benefit of both local and systemic routes, we injected 1 × 10^12^ vg intravenously (retro-orbital), and 0.5 × 10^12^ vg intramuscularly into the skeletal muscle of both hindlimb at 6 weeks of age.

**Fig. 2:**
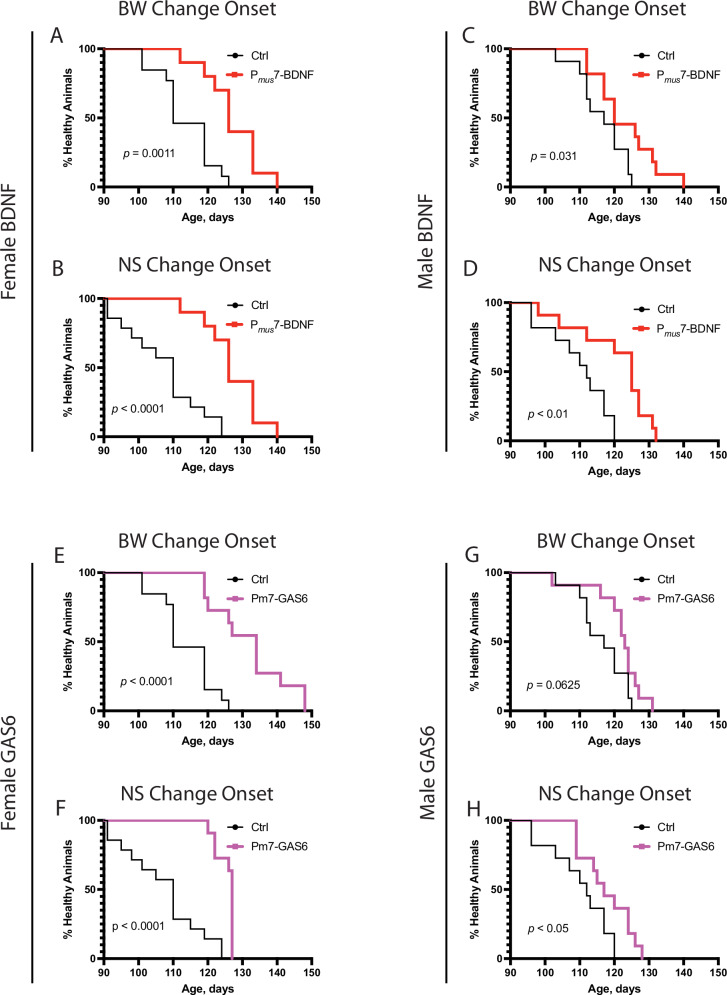
Disease onset of SOD1^*G93A*^ mice after injection of AAV-P_*mus*_7-HuBDNF-teLuc and AAV-P_*mus*_7-HuGAS6. Male and female SOD1^G93A^ mice were injected with AAV-P_*mus*_7-teLuc, AAV-P_*mus*_7-HuBDNF-teLuc, and AAV-P_*mus*_7-HuGAS6 viruses respectively. Disease onsets were determined by the age of bodyweight decrease and neurological score increase. **A**, **B** Age of disease onset in female BDNF-treated mice based on body weight and NS changes. **C**, **D** Age of disease onset in male BDNF-treated mice based on body weight and NS changes. **E**, **F** Age of disease onset in female GAS6-treated mice based on body weight and NS changes. **G**, **H** Age of disease onset in male GAS6-treated mice based on body weight and NS changes. **D** Age of disease onset in male mice based on neurological score changes. P values were determined by log rank test.

After virus injection, animal bodyweight and neurological score changes were monitored and recorded. Increases in NS score was significantly delayed in BDNF female mice compared with controls (Fig. 2B; 129.3 versus 103.2 days, respectively; *p* < 0.001). Male BDNF mice also showed significantly delayed NS changes compared to the control group (Fig. 2D; 120.5 versus 110.1 days, respectively; *p* < 0.01), although the improvement was less than that observed in female mice. Weight loss is another sign of disease onset in transgenic mice. We found that BDNF treated female mice experienced delayed body weight loss compared to control (Fig. 2A; 127 versus 114.5 days, respectively; *p* < 0.001). Again, in male BDNF treated mice, the onset of weight loss was delayed (Fig. 2C;123.1 versus 116.4 days, respectively), but less so than in female mice.

Expression of GAS6 also delayed disease onset in a similar manner. In female mice, the average NS change was delayed by GAS6 treatment (Fig. 2F; 127 versus 114.5 days, respectively; *p* < 0.0001). Again, the average NS change in male mice was delayed (Fig. 2H; 117.7 Versus 110.1 days, respectively; *p* < 0.05) but less so than observed in female mice. The average body weight change in female GAS6 treated mice was also significantly delayed (Fig. 2E; 131.8 versus 114.5, respectively; *p* < 0.0001); similarly, the time of body weight change was also delayed in male GAS6 group compared to control (Fig. 2G; 121.5 versus 116.4 days, respectively).

Based on these observations, both BDNF and GAS6 significantly delayed disease onset, as measured with both NS changes and bodyweight loss in female mice. Male mice showed significantly delayed NS change, but not the delay in weight loss compared to the control group.

### The effects of BDNF and GAS6 expression in skeletal muscle on disease progression

Our data showed that muscle-specific expression of both BDNF and GAS6 treatment delayed disease onset in ALS transgenic mice. To examine the effect of these trophic factors on disease progression, the rate of deterioration in neurological score was monitored through the course of disease, from onset until termination. We used scales from 0 (pre-symptomatic) to 4 (considered moribund and requiring euthanasia) [40]. To evaluate the locomotor performance, rotarod tests were also conducted.

**Fig. 3:**
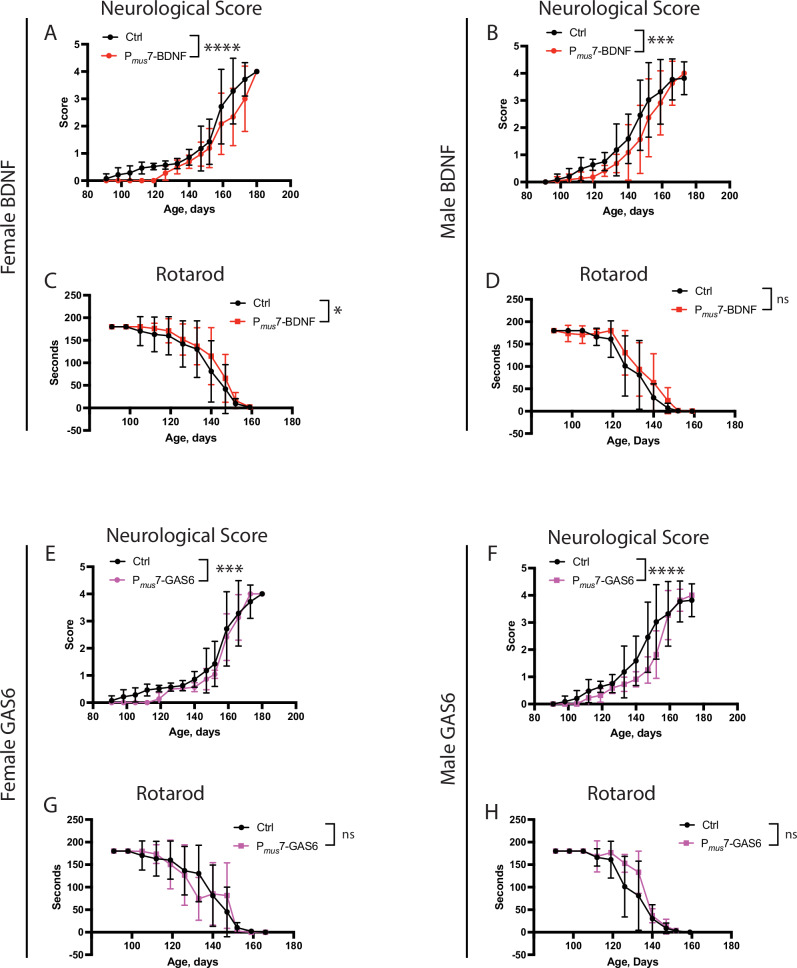
Disease progression of SOD1^*G93A*^ mice after injection of AAV-P_*mus*_7-HuBDNF-teLuc and AAV-P_*mus*_7-HuGAS6. Male and female SOD1^G93A^ mice were injected with AAV-P_*mus*_7-teLuc, AAV-P_*mus*_7-HuBDNF-teLuc, and AAV-P_*mus*_7-HuGAS6 viruses respectively. NS and rotarod test were recorded each week after virus injection. **A**, **B** NS changes during disease progression in BDNF-treated male and female mice. **C**, **D** Rotarod performances during disease progression in BDNF-treated male and female mice. **E**, **F** NS changes during disease progression in GAS6-treated male and female mice. **G**, **H** Rotarod performances during disease progression in GAS6-treated male and female mice. Values are mean ± SD. Statistics were determined by *two-way ANOVA*. * *p* < 0.05, ** *p* < 0.01, *** *p* < 0.001, **** *p* < 0.0001.

Both male and female BDNF mice showed significant reduction in disease progression as measured by NS (Fig. 3A, B). In female mice, BDNF treatment also significantly improved locomotor performance as measured by rotarod (Fig. 3C); however, no significant improvement was observed in male mice (Fig. 3D). Similarly, GAS6 treated mice exhibited a significant reduction in NS in both male and female mice (Fig. 3E, F), but improvements in rotarod performances were not significant (Fig. 3G, H).

### Effect of BDNF and GAS6 treatment in life span of transgenic mice

**Fig. 4:**
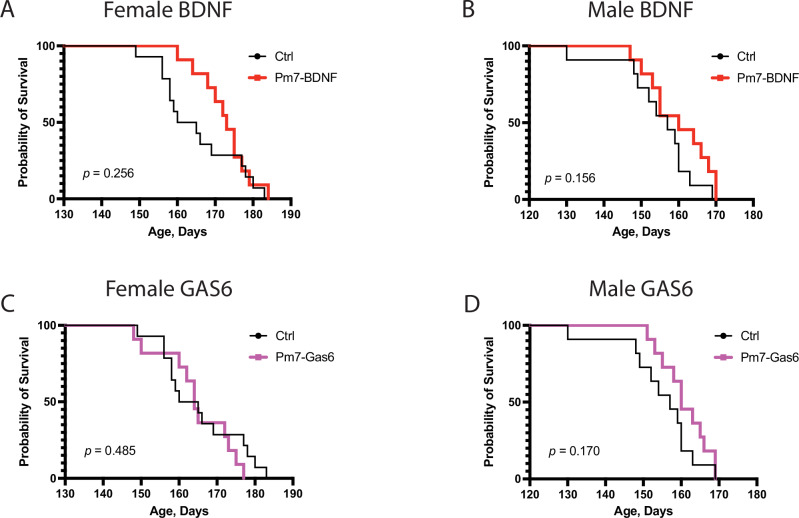
Lifespan of SOD1^*G93A*^ mice after injection of AAV-P_*mus*_7-HuBDNF-teLuc and AAV-P_*mus*_7-HuGAS6. Male and female SOD1^G93A^ mice were injected with AAV-P_*mus*_7-teLuc, AAV-P_*mus*_7-HuBDNF-teLuc, and AAV-P_*mus*_7-HuGAS6 viruses respectively. At later stage of disease progression (NS > 2), animals were monitored once to twice a day until reach the humane endpoint (NS = 4). **A**, **B** Survival curve of BDNF-treated female and male mice. **C**, **D** Survival curve of GAS6-treated female and male mice. *P* values were determined by log rank test. ns: not significant.

To evaluate if BDNF and GAS6 treatment had effects on life span, we conducted Kaplan–Meier survival analysis of the percentage of surviving animals after AAV-BDNF/GAS6 administration versus control. However, neither BDNF nor GAS6 (Fig. 4) treated mice showed statistically significant extension of life.

### Effects of BDNF and GAS6 on spinal motor neuron survival

To assess whether expression of BDNF and GAS6 delays lower motor neuron loss in ALS, mice at 75 days of age (pre-symptomatic), at 115 days of age (initiation of symptoms) and end-stage animals in control, BDNF and GAS6 treated groups (*n* = 3–5, mixed sexes) were euthanized and lumbar spinal cord were cryo-sectioned into 20 μm slices. ChAT staining was used as a motor neuron marker, and the number of ChAT positive cells in the ventral horn were quantified.

**Fig. 5:**
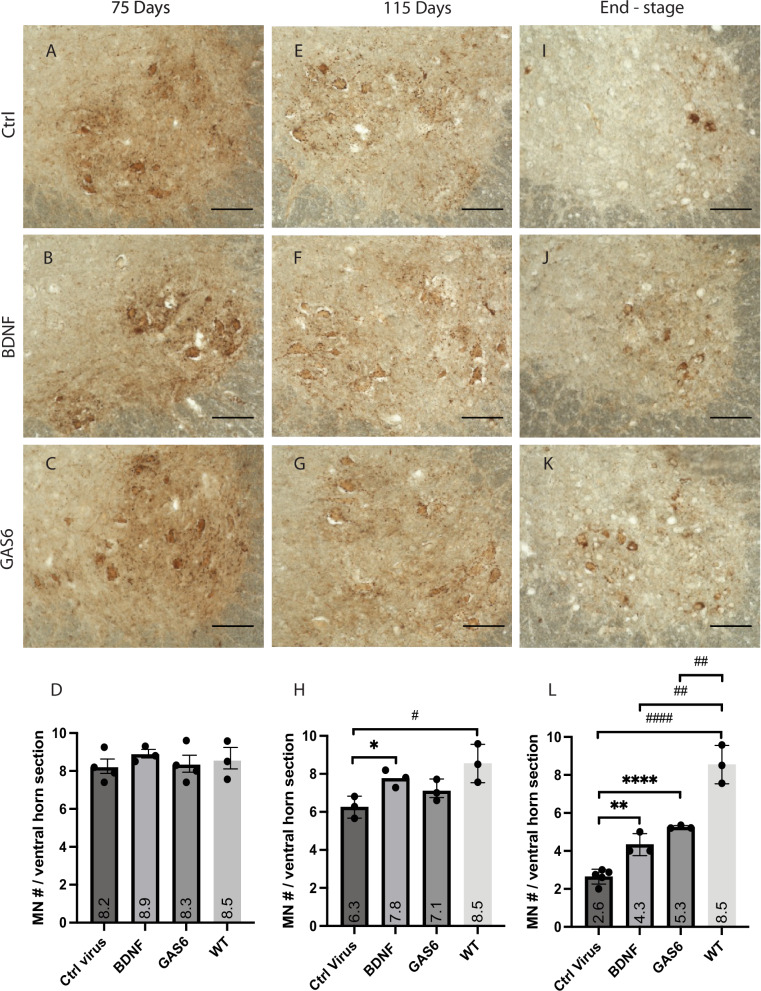
ChAT staining of lumbar spinal cord motor neuron. After virus injection at 6 weeks age, lumbar spinal cord from SOD^G93A^ mice of 75 days, 115 days age and end-stage animals (*n* = 3–4 each group, mixed sexes) were collected and cryo-sectioned into 20 μm slices. Motor neurons in the ventral horn were stained with goat-anti-ChAT primary antibody followed by peroxidase conjugated secondary antibody. Bars indicate 100  μm. **A**, **E**, **I** Motor neuron staining of different age animals from the control virus group. **B**, **F**, **J** Motor neuron staining of different age animals from AAV-P_*mus*_7-BDNF (BDNF) group. **C**, **G**, **K** Motor neuron staining of different age animals from AAV-P_*mus*_7-GAS6 group (GAS6). **D**, **H**, **L** quantifications of motor neuron in lumbar spinal cord (per ventral horn section). Values are mean ± SD. Statistics were determined by one-way ANOVA. * *p* < 0.05; ** *p* < 0.01; **** *p* < 0.0001.

At 75 days of age (Fig. 5A–D), there were no differences in motor neuron counts in each ventral horn between the wild-type, control, BDNF and GAS6 groups (8.2, 8.9, and 8.3 motor neurons, respectively). At 115 days of age, animals started to show lumbar motor neuron loss in mice injected with the control vectors; the BDNF treated 115-days old group animals had significantly more motor neurons remaining compared to the control group (7.8 versus 6.3 motor neurons, respectively; *p* < 0.05; Fig. 5E–H). When the animals reached end stage (Fig. 5I–L), an average of 2.6 motor neurons remained in the control group, indicating continued motor neuron death. BDNF and GAS6 treated animals had significantly more ChAT positive cells in the ventral horn (4.3 and 5.3 MN, respectively; *p* < 0.01, *p* < 0.0001, respectively versus control).

### Expression of BDNF and GAS6 over time in SOD1^G93A^ mice

Our data show that BDNF and GAS6 expression mediated by recombinant AAV vectors in ALS mice significantly delays the disease onset, restrains disease progression, and prevents motor neuron loss in the lumbar spinal cord. However, time to reach end-stage disease and overall lifespan were not improved significantly.

To assess the level of expression of the trophic factors in targeted tissues of treated animals, and how their levels were maintained over time, control and treated ALS mice were euthanized at 1 month or 2 months after virus injection, and at the end stage disease. Extracts of dissected heart, triceps brachii (TB), quadriceps (Quad), and brain tissues were prepared and lysed for determination of BDNF and GAS6 levels by ELISA.

**Fig. 6:**
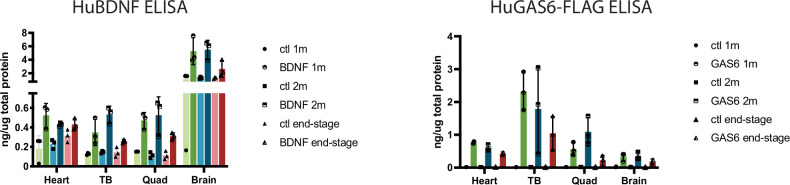
HuBDNF and HuGAS6 expression in SOD1^*G93A*^ animal tissues. Heart, TB and Quad muscles, and brain tissues from animals that received AAV-P_*mus*_7-teLuc (Ctl), AAV-P_*mus*_7-HuBDNF-teLuc (BDNF) and P_*mus*_7-HuGAS6 (GAS6) viruses were dissected after 1 month, 2 months and end-stage after virus injection (*n* = 3 each group, mixed sexes). HuBDNF and HuGAS6 levels were detected by huBDNF and Flag-tag ELISA kit. Values are mean ± SD.

The ELISA kit for human BDNF also detected endogenous mouse BDNF, because of high homology between the two species. Thus, untreated, control ALS mice tissue extracts exhibited reactivity. In BDNF-treated animals, total BDNF levels (mouse and human) were higher than control in every sample tested, reflecting expression derived from the vector. However, the virus derived protein (in treated mouse sample) appeared to decrease as the disease progressed (Fig. 6A).

Virus-coded GAS6 protein levels were specifically quantified by ELISA measurement of its FLAG tag. Again, significant amounts of protein were detected in all samples, which generally fell as disease progressed (Fig. 6B). To further validate the tissue-specific expression of this vector, GAS6-FLAG mRNA levels were measured by RT-PCR. Consistent with the protein data, mRNA expression was higher in skeletal muscles compared to heart and brain tissues (Fig. S2).

## Discussion

ALS is a progressive and fatal neurodegenerative disease [43]. With most cases being sporadic and mutations in over 30 genes linked to ALS etiology, finding an effective treatment or cure remains a significant challenge [4]. Neurotrophins and other growth factors have strong survival and protective effects on dying neurons in neurodegenerative diseases [44], making the use of trophic factors a promising strategy for both sporadic and familial ALS patients.

Various trophic factors, including CNTF, GDNF, BDNF, and IGF-1, have been investigated for ALS treatment [9]. Although some have shown benefits in alleviating symptoms and extending survival in ALS animal models, no clinical trial has yet been successful [45]. We aimed to explore new administration routes, alternative delivery vectors, and novel trophic factors to evaluate their therapeutic potential in ALS model mice.

Analysis of receptors expressed in spinal motor neurons from an RNA-seq profiling [10] revealed that Ret, Ntrk2, and Tyro3 are the most highly represented potential NTF receptors in adult mouse motor neurons The ligand for the Ret receptor, glial-derived neurotrophic factor (GDNF), has been extensively studied as a treatment in ALS. Preclinical development of GDNF has been ongoing for years, and new clinical trials were recently initiated to transplant hNPCs engineered to express GDNF in ALS patients [46]. The Trk2 receptor ligand, BDNF, one of the earliest discovered NTFs, has also been a leading candidate for ALS treatment. However, early clinical trials failed to demonstrate significant benefit, possibly due to issues with tissue permeability and sustained expression levels. Our aim has been to assess whether overexpressing BDNF in muscle tissue using an AAV vector could enhance bioavailability and achieve more sustained, long-term expression. Tyro3, a member of the TAM (Tyro3, Axl, and Mer) receptor tyrosine kinase family, plays crucial roles in hemostasis and inflammation. GAS6, the Tyro3 receptor ligand, has shown promise in recent studies for promoting microglial entry, reducing neuroinflammation in hemorrhagic mice, and alleviating white matter injury after ischemic stroke [30, 31]. However, no studies have yet explored the expression of GAS6 in ALS. Based on our RNA seq data analysis, we also chose to investigate whether ALS transgenic mice could benefit from muscle tissue-derived expression of GAS6.

NTFs have been administered through various routes in previous studies. Research has demonstrated that both intramuscular and intrathecal viral delivery of IGF-1 can significantly enhance neuroprotection in SOD1^G93A^ mice [47, 48]. Another study involving transgenic Myo-GDNF mice (which express GDNF in skeletal muscle) and GFAP-GDNF mice (which express GDNF in astrocytes) found that GDNF derived from muscle, but not from the central nervous system, delayed disease onset and extended lifespan [49]. Muscle tissue expressed GDNF can also be retrogradely transported to the spinal cord [50]. In contrast, our earlier study comparing intramuscular and intravenous injection of an inducible PHP.eB vector showed that local injection resulted in diminishing transgene expression over time, while the systemic injection group maintained sustained expression levels [42]. Therefore, we combined both systemic and local injection routes to deliver our AAV vector, aiming to ensure long-term expression and bioavailability.

In this study, we utilized an AAV9 variant, PHP.eB [38], as a therapeutic vector to express the neurotrophic factor BDNF and the TYRO3 receptor ligand GAS6, driven by muscle-specific promoter elements (*skCRM4* element and a murine Desmin promoter). The viruses were administered both intravenously and intramuscularly to SOD1^G93A^ transgenic mice. Mice treated with BDNF and GAS6 benefited from delayed disease onset and slowed disease progression as measured by NS development. We also observed that male and female mice responded differently to the treatments. Overall, both BDNF and GAS6 treatments were more effective in female mice than in males. However, male mice showed a better response to GAS6 than BDNF in terms of flattening the NS change curve and improving locomotor performance. The sex-specific differences in disease onset / progression and responses to treatments have been observed in other studies. An ALS rat model study demonstrated that the motor dysfunction started earlier in non-treated male mice than females, but the progression was similar in both genders [51]. Another mouse model study of ALS similarly found that the females survive significantly longer than males [52]. Veldink et. al. studied the effect of exercise on the phenotype of transgenic ALS mice. They observed that male mice had earlier disease onset, shorter survival than female mice; and found that exercise successfully delayed disease onset in females rather than males [53]. In contrast, a study in which AAV2-IGF-1 was delivered into the spinal cord of transgenic ALS mice, showed a male-only effect on delayed disease onset [54]. The mechanism of the sex-different response to treatment is not clear. Potential reasons include sex hormone levels, sensitivity to neurotrophic factors, and levels of mutant SOD1 expression.

Many studies have explored the roles of brain-derived neurotrophic factor (BDNF) and its receptor TRKB in the progression of ALS by examining their expression in both mouse SOD1 mouse models and human patients. The results have been inconsistent. In the muscles of ALS mice, some studies found decreased BDNF levels [55], while others reported increases [56]. In human ALS patients, BDNF levels were elevated in muscle tissues compared to controls, and other neurotrophins such as NGF, NT3, and NT4 also showed increases at late disease stages [57]. However, BDNF expression remained unchanged in the spinal cords and cerebrospinal fluid (CSF) of both ALS patients and mice [58, 59]. Serum analyses revealed either increased BDNF levels or no significant differences in ALS patients, with some studies indicating that reduced BDNF correlated with worse functional outcomes [59–61]. TRKB/TrkB expression exhibited similar variability: it decreased in the muscles of SOD1^G93A^ mice [56], whereas in the spinal cords of ALS patients, TRKB levels were elevated, though its autophosphorylation—a marker of activation—was reduced [62]. These disparate and sometimes opposing changes in BDNF and TRKB expression suggest dysregulated neurotrophic signaling in ALS. However, the inconsistent patterns observed indicate that alterations in BDNF and TRKB levels alone do not definitively reveal whether these changes are adaptive responses to the disease or contribute to its progression.

GAS6 has been shown to participate in various physiological functions including cell migration, adhesion, growth and survival [20]. Recent studies of GAS6 were focused on its role in tumor microenvironment [23]; other findings suggest that the GAS6/TAM system plays a critical role in regulating the survival of neuronal and glial cells, particularly those involved in myelination, and in controlling the innate immune response [30, 31]. This study is the first to demonstrate the therapeutic potential of GAS6 in ALS treatment. Our findings reveal that GAS6-treated mice retained a higher number of motor neurons compared to age-matched controls, though the underlying mechanisms require further investigation. Another challenge with GAS6 is its large gene sequence, which limits space for the more efficient beta-globin poly A signal in the viral construct. Using a more compact promoter could potentially allow for higher protein expression levels and, consequently, more effective outcomes.

No significant extension of lifespan was observed in any of the NTF treated groups in our study. The recombinant protein levels in tissues indicated that NTF expression decreased over time. It is possible that the reduced expression levels in end-stage animals are due to the degeneration of skeletal muscles, a common symptom in these transgenic mice. Similar studies using AAV-mediated GDNF expression in the muscle tissue of ALS mouse model also showed limited success in alleviating the symptoms [50, 63], revealing potential limitations in using neurotrophic factors in treating ALS. Promoter methylation could also contribute to the loss of expression over time. While a study in nonhuman primates showed that AAV-mediated gene transfer does not lead to significant CpG methylation of the RSV promoter, suggesting that direct DNA methylation is not involved in repressing transgene expression [64]. Further experiments need to be conducted to rule out such possibility.

Overall, this study shows that genetically directed, muscle-specific expression of protective neurotrophic factors could be of benefit in delaying the onset or ameliorating the symptoms of ALS. Although we did not see an increase in lifespan that met our statistical standard, the implications are that improvements in health and persistence of expression could achieve clinically meaningful benefit for this potential treatment.

## Supplementary information

Supplemental Figures and Methods

Figure S1

Figure S2

Figure S3

Figure S4

## Data Availability

All data generated or analyzed during this study are included in this published article.
