# Supplementary material for: AAV-mediated BDNF and GAS6 muscle delivery delays disease onset in SOD1G93A ALS mice
Source: Gene Ther. 2025 Nov 13;33(4):462–73. doi: 10.1038/s41434-025-00577-y (PMC13472854; doi:10.1038/s41434-025-00577-y)

Figure S1. Tissue specificity of $P_{mus}7$ vector in vitro.

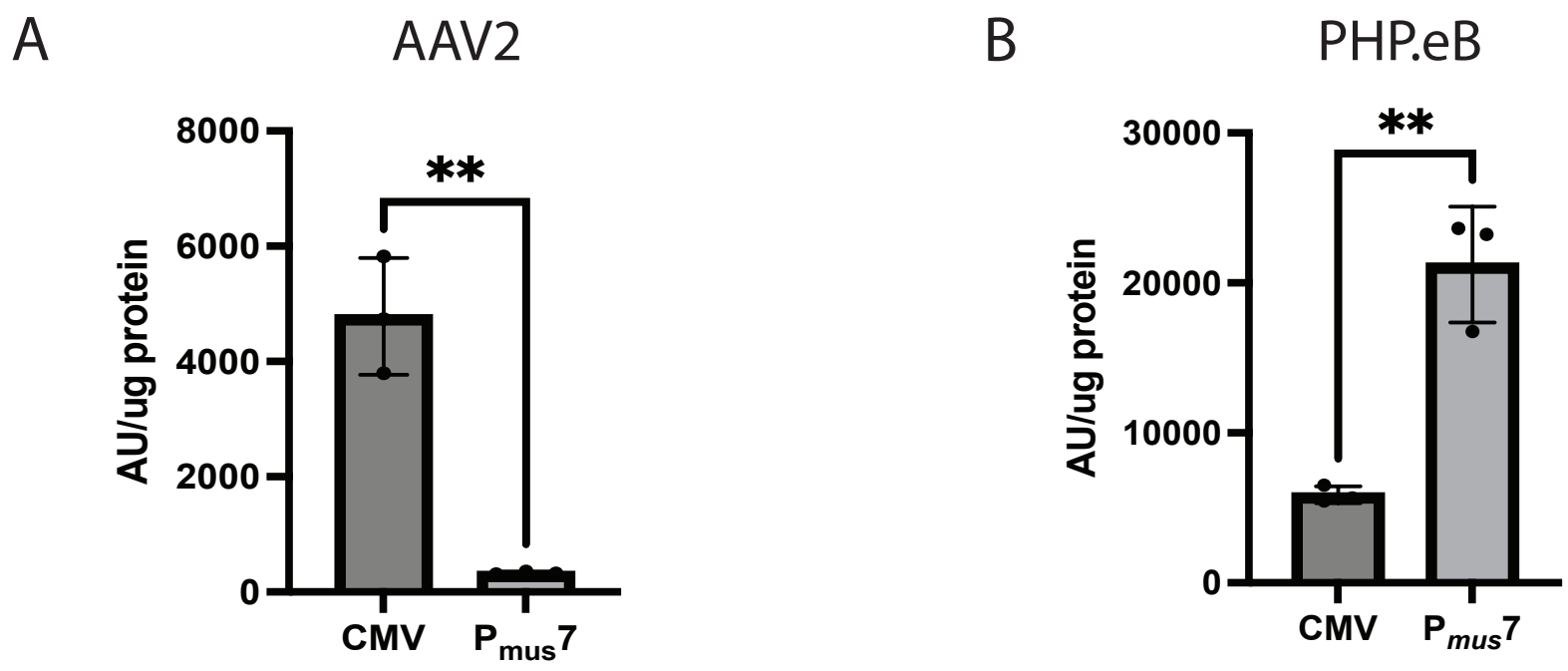

Supplement: Supplementary file 1 [file 41434_2025_577_MOESM2_ESM.pdf]
