# Supplementary material for: AAV-mediated BDNF and GAS6 muscle delivery delays disease onset in SOD1G93A ALS mice
Source: Gene Ther. 2025 Nov 13;33(4):462–73. doi: 10.1038/s41434-025-00577-y (PMC13472854; doi:10.1038/s41434-025-00577-y)
Supplement: Supplementary file 5 [file 41434_2025_577_MOESM1_ESM.docx]

**Supplementary figure legends**

**Figure S1. Tissue specificity of P*_mus_*7 vector *in vitro*.** CMV-teLuc and Pmus7-teLuc were packaged in AAV2 and PHP.eB capsids. HEK293T cells were infected by AAV2 at MOI 1000, cells were collected at day 3 post infection. C2C12 cells were infected by PHP.eB at differentiation day 4 at MOI 2000, cells were collected at day 4 post infection. Cells were then lysed for luciferase assay. (A) luciferase activity from HEK293T cells transduced by AAV2-CMV-teLuc or AAV2-P*_mus_*7-teLuc. (B) luciferase activity from differentiated C2C12 cells transduced by PHP.eB-CMV-teLuc or PHP.eB-P*_mus_*7-teLuc. Values are mean ± SD. Statistics were determined by *student’s t-test*. ** *p* < 0.01.

**Figure S2.** **Tissue specificity of P*_mus_*7 vector *in vivo.*** SOD1^G93A^ mice were injected with AAV-P*_mus_*7-GAS6. After two months, heart, TB muscle, quad muscle, and brain tissue were collected for reverse transcriptase PCR to detect exogenous GAS6-FLAG transcription levels. Values are mean ± SD. Statistics were determined by *one-way ANOVA*. * *p* < 0.05; ** *p* < 0.01.

**Figure S3. WT mouse lumbar spinal cord MN staining.** Lumbar region of the spinal cord from adult (2 months old) C57BL/6J mice were collected and cryo-sectioned onto 20 μm slices. MNs in the ventral horn were stained with goat-anti-ChAT primary antibody followed by peroxidase conjugated secondary antibody. Bars indicate 100 μm.

**Figure S4. Bodyweight change of SOD1G93A mice after AAV-Pmus7-huBDNF-teLuc and AAV-Pmus7-huGAS6.** Male and female SOD1^G93A^ mice were injected with AAV-P*_mus_*7-teLuc, AAV-P*_mus_*7-HuBDNF-teLuc, and AAV-P*_mus_*7-HuGAS6 viruses respectively. Bodyweights were recorded weekly.

**Supplementary methods**

*Luciferase assay*

Cells were lysed in NP-40 buffer (1% NP-40, 150 mM NaCl, and 50 mM Tris-Cl, pH8) supplemented with 1% (v/v) Proteinase Inhibitor Cocktail (Sigma P8340) for 30 minutes on ice, supernatants were collected after centrifuge at 1,800g for 20 minutes. Diphenylterazine (DTZ) were used as luciferase substrate. DTZ was prepared by dissolving it in an in vivo luciferase assay buffer composed of 8% glycerol (v/v), 10% ethanol (v/v), 10% Hydroxypropyl-β-cyclodextrin (m/v), 35% PEG 400 (v/v), and water to achieve a concentration of 2.5 mg/mL. For in vitro assays, the DTZ solution was further diluted in the in vitro luciferase buffer, which consisted of 1 mM Cyclohexanediaminetetraacetic acid (CDTA), 0.5% Tergitol NP-40, 0.05% antifoam 204, 150 mM KCl, 100 mM MES (pH 6.0), and 35 mM thiourea. To conduct bioluminescence measurements in 96-well plates, 20μL of cell or tissue lysates were mixed with 100μL of DTZ in the in vitro buffer. Each sample was analyzed in triplicate.

*RNA Extraction and RT-PCR*

Heart, triceps branchii (TB), quadriceps (Quad) muscles, and brain tissues were dissected from both male and female animals after 1 month of virus injection. Total RNA was extracted and purified using TriZol Reagent (Invitrogen #15596026) followed by PureLink RNA Mini Kit (Invitrogen #12183020). cDNA was synthesized by SuperScript III (Invitrogen #180180-051). RT-PCR was performed with PowerSYBR Green PCR Master Mix (ThermoFisher 4367659) on the QuantStudio qPCR systems. Primer used were: Gas6_flag-F2: 5’CTGCATGACCCTGGAAGTGA3’ and Gas6_flag-R1: 5’CTTGTAATCGATGTCGTGATCCT3’.
